# Predicting age from the transcriptome of human dermal fibroblasts

**DOI:** 10.1186/s13059-018-1599-6

**Published:** 2018-12-20

**Authors:** Jason G. Fleischer, Roberta Schulte, Hsiao H. Tsai, Swati Tyagi, Arkaitz Ibarra, Maxim N. Shokhirev, Ling Huang, Martin W. Hetzer, Saket Navlakha

**Affiliations:** 1 Integrative Biology Laboratory, The Salk Institute for Biological Studies, La Jolla, CA 92037 USA; 2 Molecular and Cell Biology Laboratory, The Salk Institute for Biological Studies, La Jolla, CA 92037 USA; 3 Molecular Stethoscope Inc., San Diego, CA 92121 USA; 4 Bioinformatics Core, The Salk Institute for Biological Studies, La Jolla, CA 92037 USA

**Keywords:** Biological age, Skin fibroblasts, Machine learning, Ensemble classifiers, RNA-seq, Aging, Biomarker

## Abstract

Biomarkers of aging can be used to assess the health of individuals and to study aging and age-related diseases. We generate a large dataset of genome-wide RNA-seq profiles of human dermal fibroblasts from 133 people aged 1 to 94 years old to test whether signatures of aging are encoded within the transcriptome. We develop an ensemble machine learning method that predicts age to a median error of 4 years, outperforming previous methods used to predict age. The ensemble was further validated by testing it on ten progeria patients, and our method is the only one that predicts accelerated aging in these patients.

**Electronic supplementary material:**

The online version of this article (10.1186/s13059-018-1599-6) contains supplementary material, which is available to authorized users.

## Background

There is a marked heterogeneity in human lifespan and health outcomes for people of the same chronological age. Thus, one fundamental challenge is to identify molecular and cellular biomarkers of aging that could predict lifespan and be useful in evaluating lifestyle changes and therapeutic strategies in the pursuit of healthy aging. Here, we developed a computational method to predict biological age from gene expression data in skin fibroblast cells using an ensemble of machine learning classifiers. We generated an extensive RNA-seq dataset of fibroblast cell lines derived from 133 healthy individuals whose ages range from 1 to 94 years and 10 patients with Hutchinson-Gilford progeria syndrome (HGPS), a premature aging disease. On this dataset, our method predicted chronological age with a median error of 4 years, outperforming algorithms proposed by prior studies that predicted age from DNA methylation [1–5] and gene expression data [3, 6] for fibroblasts. Importantly, our method consistently predicted higher ages for progeria patients compared to age-matched controls, suggesting that our algorithm can identify accelerated aging in humans. These results show that the transcriptome of skin fibroblasts retains important age-related signatures. Our computational tool may also be applicable to predicting age from other genome-wide datasets.

## Results and discussion

### Large transcriptome dataset of human dermal fibroblasts

Dermal fibroblast cells are an attractive system to study human aging for several reasons. First, fibroblasts in the human skin have a low proliferative rate and therefore are likely to retain damage that occurs with age [7]. Second, fibroblasts show age-dependent phenotypic, epigenomic, and transcriptomic changes [7–12]. Third, directly reprogrammed neurons from aged fibroblasts retain age-associated transcriptomic signatures and cellular defects, such as compromised nuclear-cytoplasmic compartmentalization [13]. Fourth, fibroblast cell lines are easily obtained from non-invasive skin biopsies. Thus, dermal fibroblast transcriptomes may encode signatures of biological age that can be extracted using machine learning methods. Researchers have previously generated transcriptomic fibroblast datasets for studying human aging; however, these datasets have been limited in the number of samples collected (< 28 individuals) and because they sample only part of the human lifespan [11–14].

To overcome these limitations, we generated an extensive RNA-seq dataset from human dermal fibroblasts, which were obtained from the Coriell Institute cell repository and the Progeria Research Foundation (“Methods” section, Additional file 1: Figure S1A). The dataset includes 133 people from 1 to 94 years old with an average of 13.3 (± 6.25) individuals per decade (Additional file 1: Table S1) and 10 HGPS patients. Cell lines from healthy individuals were cultured to a mean population doubling of 10 (± 4.2) (Additional file 1: Table S2) and from HGPS patients with mean passage number of 11 (± 0.7) (Additional file 1: Table S3). Cells were then subjected to RNA-seq analysis (“Methods” section). This fibroblast dataset is unique in its coverage of individuals across a wide range of ages and thus represents a strong benchmark for validating age prediction algorithms.

### Ensemble of classifiers to predict age from fibroblast transcriptomes

We developed an ensemble machine learning method to predict chronological age given a healthy individual’s gene expression data. Each classifier in the ensemble assigns a given sample to one of a small number of age bins (classes), with each bin having a nominal width of *N* years. For example, with *N* = 20, classifier #1 in the ensemble assigns everyone 21–40 years old to the same class. Each classifier in the ensemble is trained with a different discretization of ages, such that every possible 1-year shift of the age bins exists within the ensemble. Thus, classifier #2 groups together everyone 22–41 years old into a bin, and classifier #3 groups together everyone 23–42 years old, etc. The ensemble will therefore be made up of *N* different classifiers, each one having different age bins. The ensemble as a whole will cover every possible set of age discretizations with bin width *N* and with bin boundaries staggered 1 year apart. This concept of staggered age bins is described graphically in Additional file 1: Figure S1B.

All of the classifiers of the ensemble are trained on the same dataset, but the boundaries defining the classes (age bins) are different for each classifier of the ensemble as described above. To predict the age of a test subject, the subject’s gene expression levels are input to all *N* classifiers of the ensemble. Each classifier then predicts which age range the subject belongs to. For example, classifier #1 votes for bin 21–40 years old, classifier #2 votes for bin 22–41 years old, and classifier #3 votes for bin 3–22 years old. Each year inside the age range predicted by a classifier gets one vote recorded, and votes for each year are accumulated across classifiers. In this small example, age 20 would receive 1 vote, age 21 would receive 2 votes, age 22 would receive 3 votes, age 23 would receive 2 votes, etc. The year with the most votes is declared the prediction of the ensemble for the test subject. In the event of a tie, the youngest age prediction is chosen. In the example above, the test subject will be predicted to be 22 years old.

This ensemble method is agnostic about the type of algorithm used for each classifier. We explore different algorithms in the results below.

### Predicting age from gene expression

**Fig. 1:**
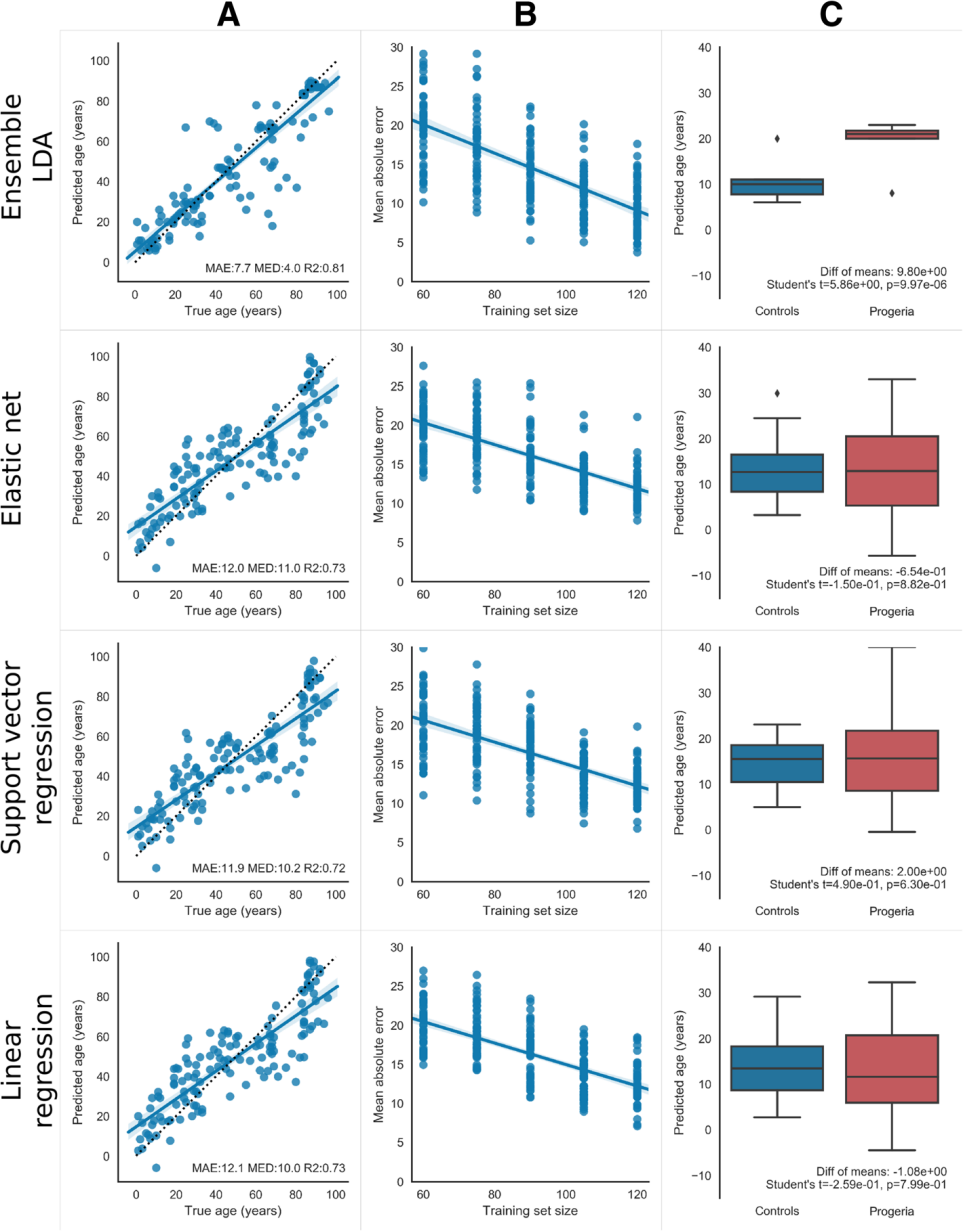
Predicting age from gene expression data. Rows from top to bottom show age prediction results for LDA Ensemble with 20-year age bins, elastic net, linear regression, and support vector regression. Model parameters are shown in Table 1. Column (A): Leave-one-out cross-validation predictions for 133 healthy individuals. Dots are plotted for each individual showing predicted age (*y*-axis) vs. true age (*x*-axis), with a line of best fit overlaid, and a shadow showing the 95% confidence interval of that line determined through bootstrap resampling of the dots. Text on the bottom of each panel shows performance metrics of mean absolute error (MAE), median absolute error (MED), and *R*^2^ goodness-of-fit for the line of best fit. The dotted line is the ideal line, where true age equals predicted age. Column (B): The effect of training set size (*x*-axis) on the mean absolute error of the ensemble (*y*-axis). The slope of the best fit line indicates the rate at which age prediction error would decrease with additional samples. Dots indicate mean absolute error from each fold of 2 × 10 cross-validation (*y*-axis) for varying sizes of random subset of the data (*x*-axis). A line of best fit and 95% confidence interval is shown. Column (C): Box plots of age predictions of progeria patients (red) and leave-one-out cross-validation predictions of age-matched healthy controls (blue). Box limits denote 25th and 75th percentiles, line is median, whiskers are 1.5× interquartile range, and dots are predictions outside the whisker’s range. The ensemble method is the only method that predicts significantly higher ages for progeria patients. Progeria patients: *n* = 10, mean ± std. of true age 5.5 ± 2.4; age-matched controls: *n* = 12, mean ± std. of true age 5.0 ± 2.9

When applied to our dataset of 133 individuals, an ensemble of linear discriminant analysis (LDA) classifiers predicted ages that differed from true chronological age by a median absolute error of 4 years and a mean absolute error of 7.7 years (leave-one-out cross-validation, Fig. 1A). This ensemble method outperformed previous algorithms [1–6] used to predict age from biomarkers, including linear regression (median = 10.0, mean = 12.1), support vector regression (median = 10.2, mean = 11.9), and elastic net regression (median = 11.0, mean = 12.0). The ensemble method also had a higher *R*^2^ of true versus predicted age compared to other algorithms: 0.81 for the ensemble versus 0.73 for linear regression, 0.72 for support vector regression, and 0.73 elastic net.

Table 1 summarizes the performance of the ensemble method using different types of algorithms and different age bin ranges. LDA provides better performance than the other algorithms we tried, including random forest, k-nearest neighbors, and Gaussian naive Bayes. Regardless of the algorithm, the error measures do not change much as bin size varies. This is particularly attractive for LDA ensembles, as age bin width is the only tunable parameter for that method.

**Table 1:** Accuracy of age prediction from fibroblast transcriptomes, for various algorithms on two datasets. Cross-validation age prediction metrics are reported for our dataset of 133 individuals between 1 and 94 years old and for dataset E-MTAB-3037 with 22 individuals from newborn to 89 years old. Metrics: mean absolute error (MAE), median absolute error (MED), and *R*^2^ goodness-of-fit for the line of best fit. Parameters shown for regression algorithms are the best ones found for reducing MAE from a grid search of the parameter space. LDA ensemble with 20-year bins (in italics) achieves a lower MAE and MED and a higher *R*^2^ than competing methods. Other window sizes (15, 25, 35) did not improve performance above that of the 20-year bin size

Algorithm	Parameters		Mean absolute error	Median absolute error	*R* ^2^
Our dataset (133 individuals)					
LDA ensemble	Age bin width = 10		9.5	4.0	0.68
	*Age bin width = 20*		*7.7*	*4.0*	*0.81*
	Age bin width = 30		8.2	4.0	0.77
Gaussian naive Bayes ensemble	Age bin width = 10	Uninformative priors	16.5	7.0	0.20
	Age bin width = 20		16.0	8.0	0.27
	Age bin width = 30		15.7	7.0	0.30
k-nearest neighbors ensemble	Age bin width = 10	Euclidean distance metric *k* = 5	22.3	14.0	− 0.19
	Age bin width = 20		19.7	11.0	0.04
	Age bin width = 30		19.7	14.0	0.09
Random forest ensemble	Age bin width = 10	n_trees = 100, min_impurity_split =2	14.2	5.0	0.38
	Age bin width = 20		11.8	5.0	0.57
	Age bin width = 30		11.8	5.0	0.55
Linear regression	N/A		12.1	10.0	0.73
Elastic net regression	Alpha = 0.1 L1/L2 ratio = 0.0		12.0	11.0	0.73
Support vector regression	Kernel = second order polynomial C = 10, epsilon = 0.05 gamma = 0.0002		11.9	10.2	0.72
E-MTAB-3037 (22 individuals)					
LDA ensemble	*Age bin width = 20*		*18.1*	*14.5*	*0.20*
Gaussian naive Bayes ensemble	Age bin width = 20, uninformative prior		36.4	39.5	− 1.47
k-nearest neighbors ensemble	Age bin width = 20, Euclidean distance metric *k* = 5		34.9	36	− 1.25
Random forest ensemble	Age bin width = 20, n_trees = 100, min_impurity_split =2		31.9	28	− 0.82
Linear regression	N/A		23.5	18.8	0.04
Elastic net regression	Alpha = 1.0 L1/L2 ratio = 0.6		20.0	18.8	0.36
Support vector regression	Kernel = second order polynomial C = 1, epsilon = 0.05 gamma = 0.0002		19.7	15.4	0.31

An important practical question is as follows: how many samples are needed to train an ensemble that can accurately predict age? Sequencing costs may be prohibitive if thousands or tens of thousands of samples are needed. To test this, we generated a learning curve for each method that depicts how age prediction accuracy changes with increasing training set size (Fig. 1B, “Methods” section). As expected, the mean absolute error decreases with more samples for all methods, and the variance of the error reduces with larger sample size. For the LDA ensemble method, extrapolating the learning curve linearly indicates that mean absolute error would drop below 5 years with an additional 19 samples and below 3 years with an additional 32 samples (Theil-Sen robust regression median slope = − 0.18, 95% confidence lower bound slope = − 0.15). It may also be the case that adding samples in particular age ranges with fewer samples will further decrease the error. This idea is supported by the anti-correlation between the number of samples in the dataset per 5 years of age range and the mean absolute errors made by the LDA ensemble in that age range (*r* = − 0.705, *p* = 0.004). While accuracy will likely asymptote as additional samples are added, these two lines of evidence suggest that further accuracy gains may be possible with feasible additional effort.

Next, we tested the general applicability and robustness of our method by using it to predict age on another publicly available RNA-seq dataset of skin fibroblasts. While we are unaware of any datasets that have comparable numbers of samples and age range, E-MTAB-3037 [13] covers a similar age range to ours (0–89 years old) but with only 22 samples. Table 1 shows that our method can predict the age of these individuals better than linear regression, support vector regression, and elastic net. As suggested by the learning curves, due to small sample size, the mean absolute error for all algorithms was higher than that with our larger dataset.

### Biological age vs. chronological age

Are the age predictions made by our method using fibroblast gene expression data also reflective of biological age? Unfortunately, the present dataset does not include health outcome data for individuals, which could be used in principle to estimate biological age. Instead, we studied the performance of the ensemble on patients suffering from Hutchinson-Gilford progeria syndrome (HGPS). HGPS provides a unique opportunity to study cellular processes in the context of what many believe is accelerated aging [15]. Mutations in the nuclear envelope protein LaminA/C result in an altered nuclear architecture, leading to a premature aging phenotype with lifespan dramatically shortened to an average of 14 years [16].

We performed RNA-seq analysis on 10 progeria patients of ages ranging from 2 to 8 years with mean age 5.5 ± 2.4 years (“Methods” section, Additional file 1: Table S3). We then compared the predicted ages for the progeria patients versus age-matched controls (i.e., each healthy person < 10 years old in our dataset, *n* = 12, mean age 5.0 ± 2.8 years) using all the algorithms previously described. Specifically, we trained each algorithm on all healthy samples in our dataset and then tested it on the progeria patients. The age predictions for age-matched controls were taken from the leave-one-out cross-validation of all 133 healthy individuals.

Our ensemble of LDA classifiers consistently predicts progeria patients as older than age-matched controls, as expected, whereas, none of the previously proposed methods predicted accelerated aging in progeria patients (Fig. 1C). To gauge significance, we performed Student’s *t* tests comparing the predicted age of HGPS patients vs. age-matched controls for each method listed in Table 1. LDA ensembles had a difference between group means that ranged from 9 to 10 years (progeria older), depending on age bin size. *p* values were highly significant, ranging from 1e−4 to 1e−5, indicating that HGPS patients were predicted significantly older than age-matched controls. Random forest and naive Bayes ensembles of various age bin sizes had a higher mean difference between groups (15 to 24 years, progeria older) but had lower *p* values ranging from 1e−3 to 1e−2. The other ensembles and all regression methods had only a small, insignificant difference between group means, often with controls older than patients, and *p* values around 1e−1. Thus, only the LDA ensemble method predicts HGPS patients as significantly older than age-matched controls after Bonferroni correction, suggesting that this technique is a better gauge of biological age compared to other methods.

## Discussion

We developed an ensemble machine learning method that combines classification into age ranges with voting-based regression across classifiers. Our method predicts age from gene expression data in cultured human fibroblasts more accurately than regression algorithms, such as ElasticNet, which have been previously used to predict age from transcriptomic, epigenetic, and other kinds of biomarkers [1–6]. When applied to our 133 sample dataset, the ensemble method produced a 4-year median error and a 7.7-year mean absolute error during leave-one-out cross-validation, which is comparable to the 7.8-year mean error produced from transcriptomic data in the blood [3]. Our results are not far off the performance of Horvath’s [1] DNA methylation clock (3.9-year median absolute error from 7800 samples) or Putin’s [17] deep learning method using blood cytology and chemistry (5.5-year mean absolute error from 56,000 samples), but uses one to two orders of magnitude fewer samples. This level of prediction performance using few samples is encouraging, yet the strength of the DNA methylation clock is the large numbers of studies and samples that have supported its use as an age predictor. While the learning curve (Fig. 1B) suggests performance of our method will increase with additional samples, further work is required to test this. Furthermore, the ensemble method predicted HGPS patients as significantly older than age-matched controls, suggesting that the predictions made may reflect biological age rather than chronological age. In contrast, the regression algorithms did not make this prediction.

Why is the ensemble method so effective at predicting age from gene expression in fibroblasts? A necessary and sufficient condition for an ensemble to be more accurate than any of its individual classifiers is that those classifiers are accurate and diverse [18]. An accurate classifier has a lower error rate than uniform random guessing, and diverse classifiers make different errors when generalizing to new data. One taxonomy of ensemble learning [19] suggests that there are different ways to create classifier diversity, including manipulating the training examples (e.g., bootstrap aggregation) and the input features (e.g., sub-setting genes) or by injecting randomness (e.g., through random number generation for non-deterministic algorithms). Staggered age bins perform sample manipulation by grouping together different sets of subjects as belonging to the same age class for different classifiers of the ensemble. It is possible that this ensemble method with staggered discretizations could be generalized to other problems that involve prediction of smooth values within finite bounds.

The ensemble with staggered age bins can be used with any kind of classification algorithm. LDA might be a particularly good choice for the ensemble because groups of genes involved in the same biological process are believed to co-vary in their expression. The LDA algorithm can exploit covariance structure to aid in classification, while its close relative naive Bayes assumes independence of the variables. Indeed, LDA had a mean error less than half that of naive Bayes, which might reflect the interdependent nature of gene expression. While it is known that the transcriptional output of individual genes can change during aging [20], it is not yet clear if covariance patterns exist in gene expression programs over human lifespan [21]. Future work with our method may add to our understanding of how gene networks vary with age.

The ensemble method has some limitations. First, the discretization of age produces clear edge effects in predictions at the youngest and oldest ends of the age spectrum, where there is systematic over- and underprediction. In contrast, regression-based techniques systematically overpredict both young and old people (c.f., Fig. 1A). Including more samples in these age ranges may help overcome this problem. Second, it may be difficult to interpret the relationship between changes in gene expression and biological aging, as predicted by the ensemble method. Understanding mechanisms of aging remains an important challenge for biomedical research and our approach provides a tool to study aging in humans. Third, the covariance estimation process employed during LDA has some drawbacks with datasets like ours. For example, it is well-known that the sample covariance matrix may not be well-conditioned or a good estimate with large numbers of variables and small numbers of samples; thus, we used Ledoit and Wolf’s technique [22] to address this problem. Because LDA involves estimating covariance matrices and performing an eigenvalue decomposition, LDA on large numbers of variables is computationally costly. Other classifier algorithms may be employed within our general ensemble framework to address some of these problems.

We noticed that the ensemble method consistently predicted some individuals as relatively older or younger than their chronological age across many bootstrap re-samplings and by almost all of the individual classifiers within the ensemble (data not shown). It is unclear if these individuals were incorrectly predicted due to poor performance of the classifier, or because these individuals had a markedly different biological age compared to their chronological age. Future studies should attempt to tease apart these differences by collecting additional health-related markers.

## Conclusions

Our dataset of fibroblast gene expression is unique in its broad coverage of healthy individuals whose ages span from 1 to 94 years. Remarkably, skin fibroblast cells that have been cultured ex vivo retained signatures that allowed us to accurately predict an individual’s age. We developed an ensemble machine learning method that predicted age to a median error of 4 years, outperforming previous methods used to predict age from genomic biomarkers. Our results suggest that skin fibroblast transcriptome data, coupled with machine learning techniques, can be a useful tool for predicting biological age in humans. Applying this approach in a longitudinal study raises the possibility of developing a monitoring and prognostic tool for aging and related diseases.

## Methods

### Cell culture

Human dermal fibroblasts documented as “apparently healthy individuals” deposited from the National Institute of Aging or the NIGMS Human Genetic Cell Repository were obtained or purchased from the Coriell Institute Cell Repository (Camden, NJ, USA). The cells were cultured in high glucose (4.5 mg/ml) DMEM (Thermo Fisher Scientific Gibco) supplemented with 15% (vol/vol) fetal bovine serum (Thermo Fisher Scientific Gibco), 1X glutamax (Gibco), 1X non-essential amino acids (Thermo Fisher Scientific Gibco), and 1% (vol/vol) penicillin-streptomycin (Thermo Fisher Scientific Gibco). HGPS patient fibroblasts were obtained from the Progeria Research Foundation and were cultured in high glucose (4.5 mg/ml) DMEM (Thermo Fisher Scientific Gibco) supplemented with 20% (vol/vol) fetal bovine medium and 1% (vol/vol) penicillin-strepomycin. All fibroblasts were cultured in DMEM unless specified and were maintained at 37 °C in a humidified incubator with 3% O2 and 7.5% CO2. Cells were passaged every 2 to 5 days at 85% confluence.

### RNA sequencing

Total RNA was extracted using 1 ml of TriZol reagent (Thermo Fisher Scientific Invitrogen) directly from the six-well plates or trypsinized and pelleted from 100-mm dishes. RNA was purified with RNeasy Mini kits with 30 min of DNase I treatment to remove genomic DNA in accordance to the manufacturer’s instructions (Qiagen). mRNA sequence libraries were prepared using the Illumina TruSeq Stranded mRNA kit with 150 ng to 1μg of total RNA. Reads were aligned to the human genome (hg19) using STAR (version 2.5.1.b) [23]. Only reads that aligned uniquely to a single genomic location were used for downstream analysis (MAPQ > 10). Gene expression (FPKM) values were calculated for annotated RefSeq genes using HOMER (version 4.9.1) by counting reads found overlapping exons [24]. As most genes typically express just one dominant isoform, the top expressed isoform was used as proxy for gene expression, resulting in 27,142 unique genes. While isoform-level quantification of expression may help better stratify age, our data is not able to address this question, as it would require deeper sequencing, paired-end library preparation, and/or long-read sequencing.

### Model fitting and evaluation

Age prediction models were fit to the FPKM data for all subjects using leave-one-out cross-validation. Model performance is reported on the average error made on the held-out sample for each validation fold.

For each cross-validation fold, a different set of genes were selected to train the model according to a fixed rule. To be included in building the model, a gene must have at least a fivefold difference in expression levels between any two samples in the training set, and at least one sample in the training set had to have an expression level > 5 FPKM for that gene. Averaged over the 133 folds of leave-one-out cross-validation, this resulted in 4852 genes used to train the model, with a standard deviation of 17, a minimum of 4755 genes, and a maximum of 4861 genes. Various other methods for gene sub-setting were implemented with little change in the age prediction results. For example, using stronger inclusion criteria (tenfold change, max FPKM > 5) tends to produce similar results in regression models, while using much weaker criteria (twofold change, max FPKM > 1) produces approximately 20–40% worse mean absolute error, as the number of genes included in the model more than doubles. After sub-setting, selected gene FPKM values were then log transformed before further use. Note that gene sub-setting is different across folds of the cross-validation to prevent overfitting, but since the same folds will be used for training each model, gene sub-setting is identical across models.

Calculation of learning curves (i.e., how training set size effects prediction accuracy) was done by creating multiple bootstrap samples of the data for each training set size under investigation using 5 × 10-fold cross-validation.

Age prediction models were fit using scikit-learn v.0.19 [25] in Jupyter notebooks v.5.0.0 [26] running python v.2.7.9. Linear regression models were fit using ordinary least squares. Elastic net models were fit using coordinate descent; regularization ratio and proportion (L1/L2 ratio and alpha) were selected using a parameter grid search with 2 × 10-fold cross-validation on the dataset. Support vector regression models were fit using libsvm, for both polynomial kernels up to degree four and radial basis kernels. Parameters for kernel, degree, C, gamma, and epsilon were selected by 2 × 10-fold cross-validation grid search. For the ensemble of classifier method, parameter values (which included any classifier-specific parameters, as well as the ensemble’s age bin size parameter) were selected using a parameter grid search with 2 × 10-fold cross-validation. Linear discriminant analysis was performed using the eigenvalue decomposition solver and Ledoit-Wolf shrinkage of the covariance matrix [22]. Naive Bayes, k-nearest neighbors, and random forest algorithms were implemented with their default settings in scikit-learn. In all cases, after grid search was used to select the best model parameters, model performance was evaluated separately using leave-one-out cross-validation to generate the results in Fig. 1 and Table 1. As a further check against overfitting, we performed 10× random shuffles of 50%-50% training-test split (i.e., twofold cross-validation). The results, shown in Additional file 1: Figure S2, show that the ensemble LDA method continues to outperform the other methods on our dataset. For a detailed mathematical treatment of the classification and regression algorithms used here, see [27].

We downloaded and aligned fastq files using the same methods described in the RNA sequence section above to produce FPKM for E-MTAB-3037 [13] (22 people, 0–89 years old). We then used the same model fitting procedures described above for our data. The ages of the samples in E-MTAB-3037 could be predicted by various methods, with the ensemble LDA method having the lowest mean/median absolute errors (Table 1).

## Additional files

Additional file 1: Supplementary Material. (DOCX 2383 kb)

Additional file 2: Review history. (DOCX 476 kb)
