# Supplementary material for: Predicting age from the transcriptome of human dermal fibroblasts
Source: Genome Biol. 2018 Dec 20;19:221. doi: 10.1186/s13059-018-1599-6 (PMC6300908; doi:10.1186/s13059-018-1599-6)

Supplementary Information

Table S1. Sample count per decade of cell lines.

Table S2. Human dermal fibroblast cell lines used in this study. Fibroblast lines were from the Coriell Institute cell repository. All cells employed were documented as from an “apparently healthy individual”. Population density level (PDL) is a measurement for the age of cells in culture calculated by 3.3(log (total viable cells at harvest/total viable cells at seed)). Passage (PASS) number is the number of times the cells have been split in culture. Cell lines without PDL and/or PASS information from Coriell are tracked as PDL+ and PASS + after arrival.

Table S3. Hutchinson-Gilford Progeria Syndrome (HGPS) patient fibroblast cell lines. Cell lines come from either Coriell Institute or Progeria Research Foundation. Identifications (ID) prefaced with PRF or HGADFN are from Progeria Research Foundation. ID prefaced with AG is from Coriell Institute.

| **Coriell/PRF ID** | **Age** | **PASS** | **Condition** |
| --- | --- | --- | --- |
| AG11513 | 8yr | Unknown | HGPS |
| PRF167 | 8yr | 13 | HGPS |
| HGADFN188 | 2yr3mos | 11 | HGPS |
| HGADFN127 | 3yr9mos | 12 | HGPS |
| HGADFN164 | 4yr8mos | 12 | HGPS |
| HGADFN169 | 8yr6mos | 11 | HGPS |
| HGADFN178 | 6yr11mos | 12 | HGPS |
| HGADFN122 | 5yr0mos | 12 | HGPS |
| HGADFN143 | 8yr10mos | 12 | HGPS |
| HGADFN367 | 3yr0mos | 11 | HGPS |

Figure S1. **Overview and computational pipeline.** (A) Fibroblast samples from 133 healthy individuals and 10 Progeria patients were obtained from the Coriell biobank, cultured, and sequenced. (B) Illustration of the ensemble of classifiers used to predict age. Each classifier in the ensemble is trained with a different discretization of age ranges into bins using a sliding window. For example, Classifier 1 has five age range bins illustrated by the five rectangles. Classifier 2 has six bins, with the boundary of each bin shifted by one year from the previous classifier. Each classifier provides a single vote of the age range for the test sample. All ages in that range receive a single vote. Votes are then summed over all classifiers, and the age that receives the most votes is predicted as the age of the held-out sample. Ensembles are trained with leave-one-out cross-validation (STAR Methods). Shown is the ensemble with an age bin width of N=20 years. Note that for any given dataset where the age difference between the oldest and youngest individuals is M years, M/N may not be an integer number; hence the youngest and oldest bins may have age ranges covering less than N years, as shown in panel B.


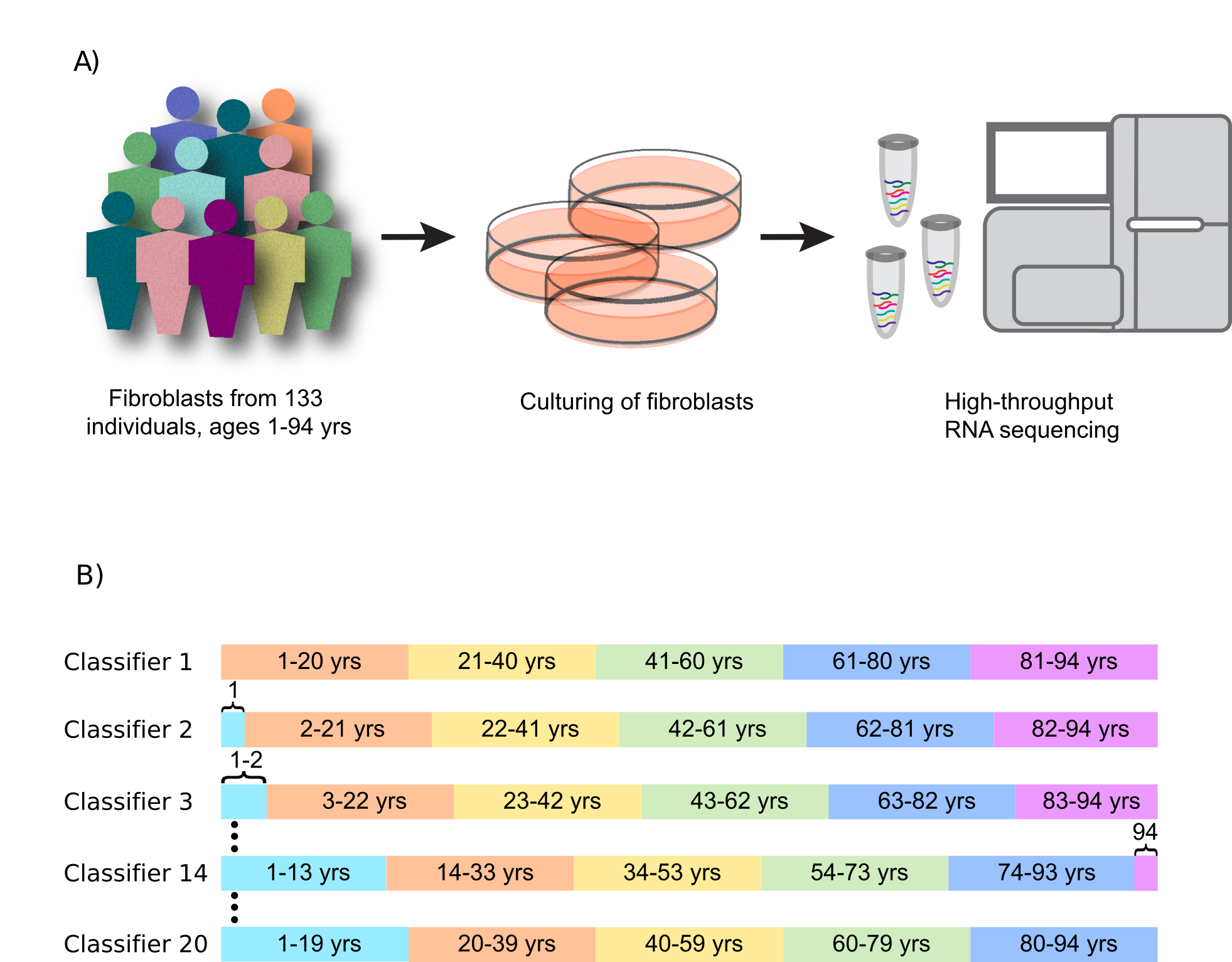


Figure S2. Prediction results from performing 2-fold cross-validation (50% train, 50% test). Blue dots show true (x-axis) and predicted (y-axis) ages for samples in the test set. To eliminate random sample effects, the train-test split was done with 10 random shuffles of the data. Consistent with our previous results when doing leave-one-out cross-validation in Figure 1 and Table 1, the LDA ensemble has the lowest mean and median absolute errors (MAE and MED, as noted on plots) between predicted and true ages. Dotted line shows where the line of zero prediction error lies; blue solid line is the line of best fit through the dots.


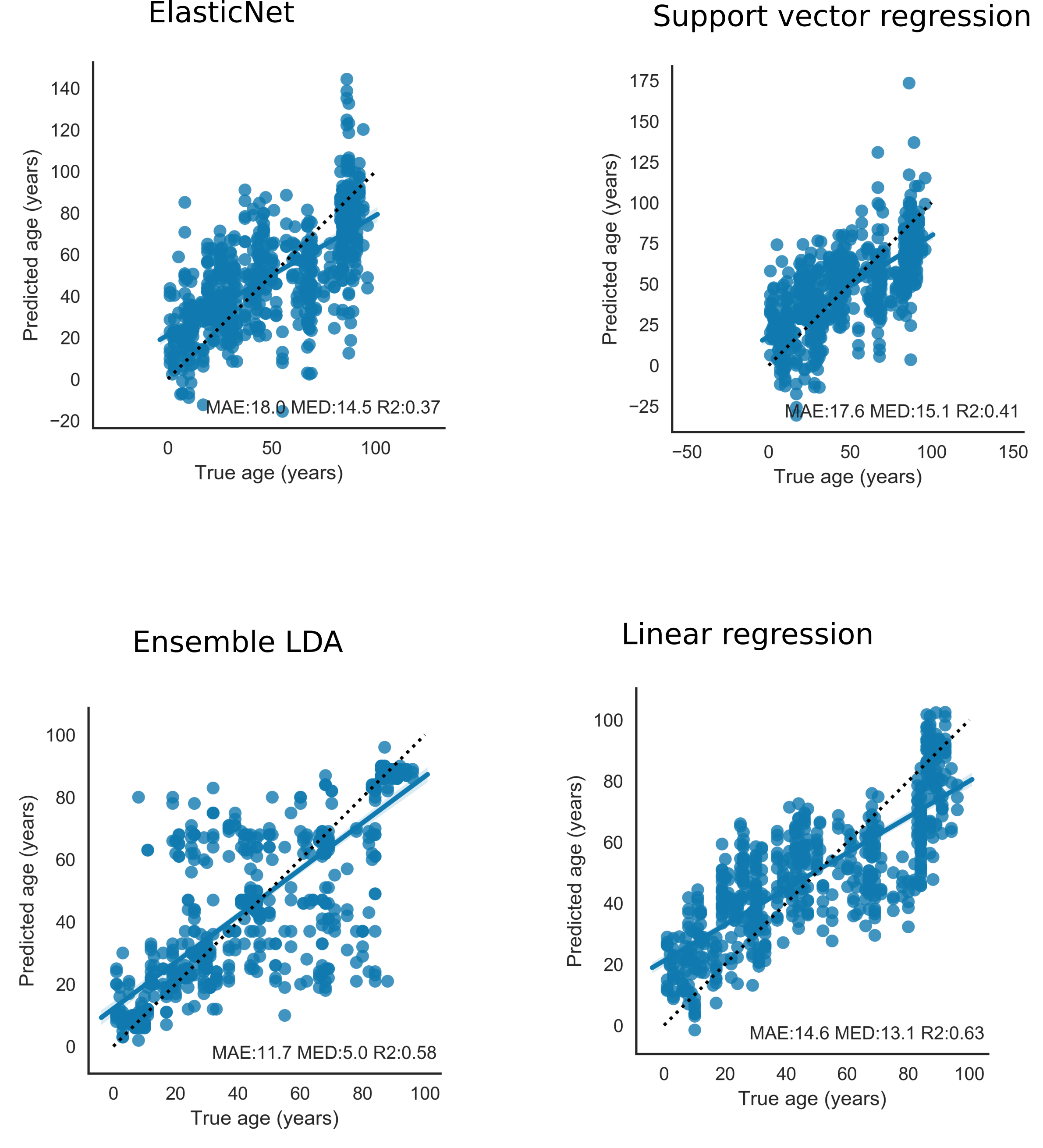


Figure S3. A different view of the results from Figure 1C, showing the age prediction of each subject for the four age prediction methods. The dotted line indicates where the points would lie if prediction error was zero. Progeria patients are plotted in red, and the age-matched controls in blue. Predictions were formed by training on all 133 samples from healthy subjects, and testing the resulting methods on the Progeria patients.


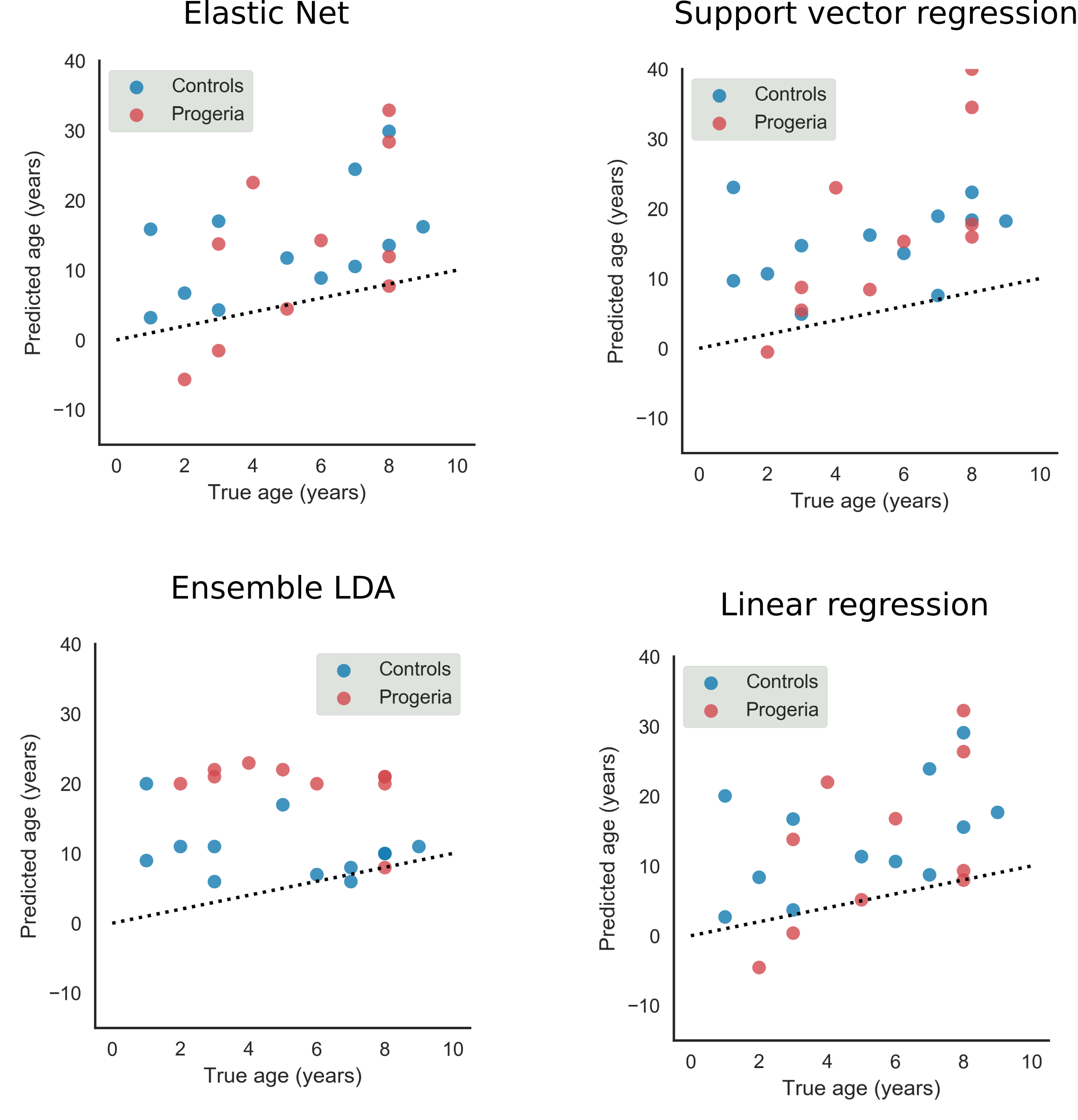


Figure S4. The relationship between age prediction error and sample density in that age range. In age ranges where sample density (blue) is lowest, ensemble LDA has the highest mean error. Pearson correlation (p) between the two values is reported in the plot. The regression methods do not have as strong a relationship between error and sample density (p roughly -0.30 compared to -0.67 for ensemble LDA).


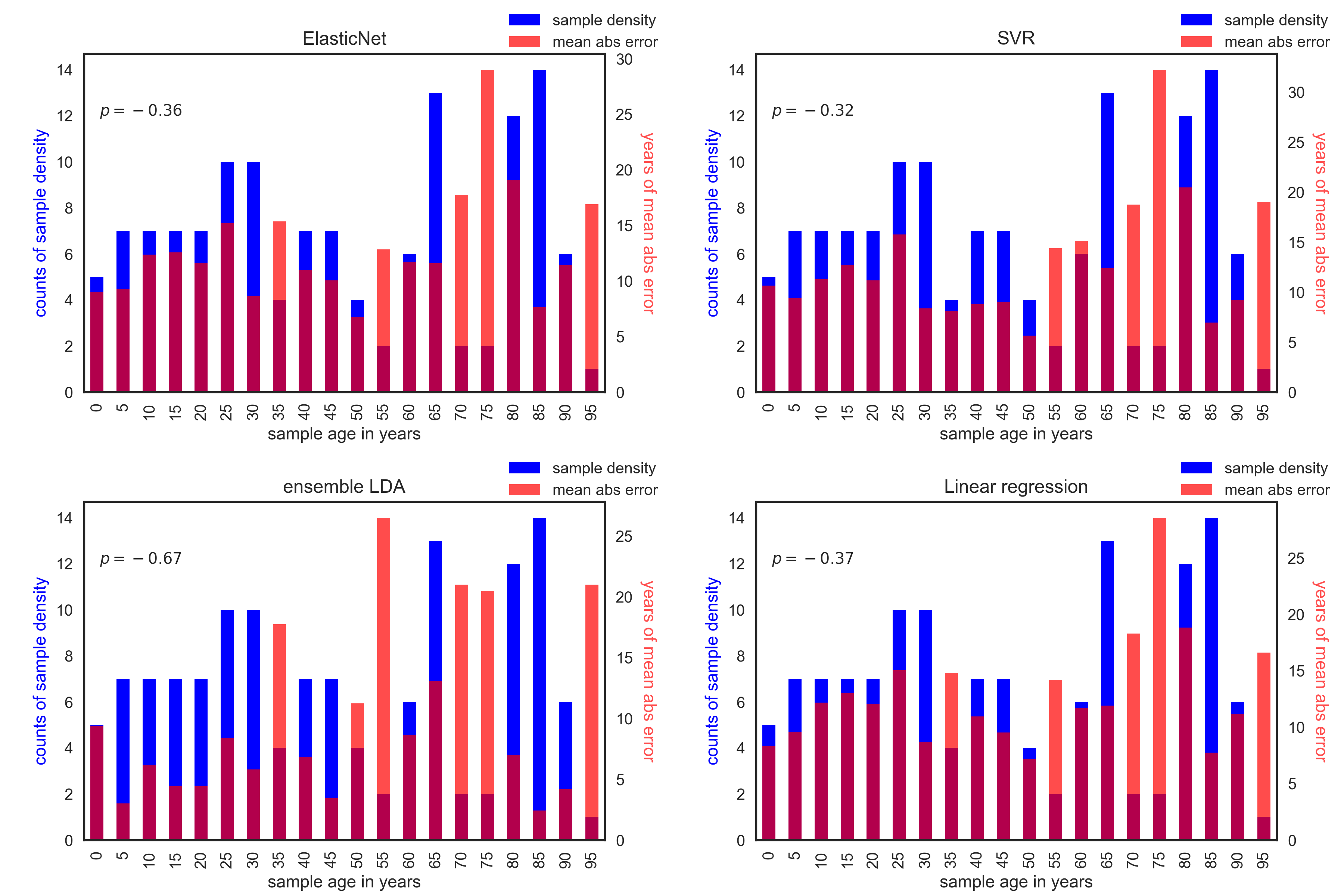

Supplement: Supplementary file 1 — Supplementary Material. (DOCX 2383 kb) [file 13059_2018_1599_MOESM1_ESM.docx]
