# Supplementary material for: Predicting age from the transcriptome of human dermal fibroblasts
Source: Genome Biol. 2018 Dec 20;19:221. doi: 10.1186/s13059-018-1599-6 (PMC6300908; doi:10.1186/s13059-018-1599-6)
Supplement: Supplementary file 2 — Review history. (DOCX 476 kb) [file 13059_2018_1599_MOESM2_ESM.docx]

Review History

**First round of review**

**Reviewer 1**

**Are the methods appropriate to the aims of the study, are they well described, and are necessary controls included?**
Yes

**Are the conclusions adequately supported by the data shown?**

No, please see my review for details.

**Are sufficient details provided to allow replication and comparison with related analyses that may have been performed?**

Yes

**Does the work represent a significant advance over previously published studies? Please explain.**

Probably yes, please see my review for details.

**Is the paper of broad interest to others in the field, or of outstanding interest to a broad audience of biologists?**

No

Comments to authors:

In this work, the authors develop a method to predict age from gene expression profiles of human skin cells. Developing biomarkers of aging is a very timely topic and the approach employed by the authors is potentially useful, although I have a few concerns about the work, as detailed below.

I didn't quite understand the ensemble of classifiers.

As far as I can tell, the authors employ different age classifications, presumably reaching different gene combinations

, and then calculate a sort of average of all of the classifiers. I thought this was a little confusing, and it also makes me wonder how can others use this method and classifiers to predict age for their samples? I also wonder whether the genes used in the classifications could provide any insight about aging. Is the data on genes used in the predictions provided? I think having some sort of idea of the type of gene expression signatures of aging derived would be interesting, in particular since, as the authors point out, it is remarkable that cultured cells ex vivo retained signatures of the individual's age.

Then as far as I understand the authors test various methods to predict the classifiers and settle on one using cross validation. Then the authors emphasize the ability of their method to predict higher ages for progeria patients. However, I don't think progeria is universally accepted as accelerated aging, so such validation will not be convincing for everyone.

Related to the above, I also wonder about problems of overfitting, and whether other forms of validation would strengthen the work. For example, could the authors maybe split their data in half and then test how accurate the classifiers developed for half of the data are for the other half? Obviously it would be ideal to have another data set the authors could apply their method to, but I'm not sure this exists.

In the same paragraph the authors write: "This method predicts age from gene

expression data in human fibroblasts more accurately than previous methods used to predict age

from transcriptomic or epigenetic biomarkers [1-6]." Then write: "Our results are not far off the performance of Horvath's [1]". This seems like a contradiction, and overall I think the authors are overselling their findings in the discussion and should tone down their conclusions.

For instance, the authors write that: "our method was the only one that correctly predicted accelerated aging in these patients." As far as I can tell the authors didn't test other methods, like epigenetic clocks, so I don't understand how can they make such claim.

I couldn't find information on how long and how many passages were cells cultured before the gene expression profiling.

Please include page numbers in future manuscripts, to make it easier for reviewers to refer to specific sections in the manuscript.

Overall, I think this is an interesting and timely work, but some aspects may still be clarified and I think the conclusions should be toned down. Some additional validation would further strengthen the work. It is possible that some of my comments reflect misunderstandings of mine. If so then I would suggest that the authors use my misunderstandings as an indication that such points might be made clearer in the manuscript.

It my usual policy to reveal my identity to the authors: Joao Pedro de Magalhaes.

**Reviewer 2**

**Are the methods appropriate to the aims of the study, are they well described, and are necessary controls included?**
Yes

**Are the conclusions adequately supported by the data shown?**

Yes

**Are sufficient details provided to allow replication and comparison with related analyses that may have been performed?**

No. In order to achieve this goal the authors need provide the input matrix and the scripts.

**Does the work represent a significant advance over previously published studies? Please explain.**

Yes

**Is the paper of broad interest to others in the field, or of outstanding interest to a broad audience of biologists?**

No

**Comments to author**

In this manuscript the authors have developed a computational method that can predict the biological age of an individual from RNA-seq data of skin fibroblasts. Their method could predict human age to a median error of 4 years, outperforming prior algorithms. Their method is capable to predict accelerated aging from transcriptomic data of 10 Progeria patients.

I suggest acceptance with minor revision. My questions are below.

What is the reason that different gene sets are used by different methods? By intuition, the same input (matrix of FPKM values for genes on the row and samples on the column) will make the comparison between methods on a fair basis.

why is the 20-year window better? Has the author examined other window sizes in addition to 10,20, and 30?

For the 22 test individuals in table 1, why aren't all other methods presented for this test?

I suggest the authors also provide a scatter plot like Fig1A for the data in Fig1C. Maybe they can arrange this figure in the supplementary.

Why is LDA better? Although the authors mentioned "The efficacy of our ensemble method can be attributed to several features", that paragraph is not clearly explaining the "several features" and why those features make LDA better. Some further explanation is suggested but not required or absolutely necessary.

Not sure whether it is due to the big size of the dots in the Fig1A, there seem to be a large deviation for the ages between 50~70ish for the LDA method while other methods could more accurately predict these ages. Could the authors explain this phenomenon? Is it possibly due to the different gene sets used for different methods? It's easy to decline a method if it predicts a 70-year old human being to be only 20-year old!! From these scatter plot, I would use LDA for most of the predictions but use the average of the other three methods if they are concordantly far away from the LDA prediction.

The authors did not explain why "The top expressed isoform was used as proxy for gene expression" while a common usage for gene expression to count all the reads on exons and compatible isoforms.

Lastly, and most importantly, I think the authors should present the input FPKM matrices and downstream scripts for each prediction as additional files such that readers could learn the details of this manuscript.

**Authors Response**

**Point-by-point responses to the reviewers’ comments:**

**Reviewer #1**

1. I didn't quite understand the ensemble of classifiers. As far as I can tell, the authors employ different age classifications, presumably reaching different gene combinations, and then calculate a sort of average of all of the classifiers. I thought this was a little confusing, and it also makes me wonder how can others use this method and classifiers to predict age for their samples?

*We apologize for not making our method clearer. To alleviate confusion, we rewrote the description of the ensemble method (pp 2-3, section “Ensemble of classifiers to predict age from fibroblast transcriptomes”) to include more concrete examples. The new text is copied below:*

*“Each classifier in the ensemble assigns a given sample to one of a small number of age bins (classes), with each bin having a nominal width of N years. For example, with N=20, classifier*

*#1 in the ensemble assigns everyone 21-40 years old to the same class. Each classifier in the ensemble is trained with a different discretization of ages, such that every possible 1-year shift of the age bins exists within the ensemble. Thus, classifier #2 groups together everyone 22-41 years old into a bin, and classifier #3 groups together everyone 23-42 years old, etc. The ensemble will therefore be made up of N different classifiers, each one having different age bins. The ensemble as a whole will cover every possible set of age discretizations with bin width N and with bin boundaries staggered 1-year apart. This concept of staggered age bins is described graphically in Supplementary Figure S1B.*

*All of the classifiers of the ensemble are trained on the same dataset, but the boundaries defining the classes (age bins) are different for each classifier of the ensemble as described above. To predict the age of a test subject, the subject’s gene expression levels are input to all N classifiers of the ensemble. Each classifier then predicts which age range the subject belongs to. For example, classifier #1 votes for bin 21-40 years old, classifier #2 votes for bin 22-41 years old, and classifier #3 votes for bin 3-22 years old. Each year inside the age range predicted by a classifier gets one vote recorded, and votes for each year are accumulated across classifiers. In this small example, age 20 would receive 1 vote, age 21 would receive 2 votes, age 22 would receive 3 votes, age 23 would receive 2 votes, etc. The year with the most votes is declared the prediction of the ensemble for the test subject. In the event of a tie, the youngest age prediction is chosen. In the example above, the test subject will be predicted to be 22 years old.”*

*We also replaced several instances (mostly on p.3, but also one each on p.4 and p.5) of confusing wording, where we referred to both a member of the ensemble and the algorithm used as a “classifier”. We chose to reserve “classifier” to describe a member of the ensemble, and “algorithm” to describe the kind of classifier being employed (LDA, Naive Bayes, etc).*

*To help further clarify for readers, we will seek Editorial approval for adding Supplementary Figure S1 (a graphical depiction of the method) to the main text; we initially left it in the Supplementary because the short report format is limited to 2 figures/tables, which we used to focus on age prediction results.*

*Re: “how can others use this method and classifiers?” This is an important point, and we sought several ways to make our method as easy to use as possible. First, we released the software implementing our ensemble method and our entire dataset as open source/access (see section “Availability of data and material”). Second, the software is built from a commonly used machine learning library, called scikit-learn. The main paper related to this library (Pederagosa, 2011) has more than 11,000 citations, which suggests that there are many programmers who are familiar enough with this library to use, evaluate, and extend our software for different applications. Third, the software we wrote is intended to be highly modular so it can be used for different input data. Additionally, the modification of parameters for learning and gene subsetting requires only minor changes.*

2. I also wonder whether the genes used in the classifications could provide any insight about aging. Is the data on genes used in the predictions provided? I think having some sort of idea of the type of gene expression signatures of aging derived would be interesting, in particular since, as the authors point out, it is remarkable that cultured cells ex vivo retained signatures of the individual's age.

*We agree with the reviewer that there is great interest in understanding the mechanisms of aging. The strength of our study lies in the ability to identify predictive gene expression signatures of aging in human skin fibroblasts without prior knowledge about the cellular and molecular underpinnings of aging. It is likely that the observed changes in gene expression are the result of epigenetic modifications that are acquired as the individual is aging. Since our goal here is to create a diagnostic for aging, we did not focus on teasing apart correlation from causation. Any correlative result, no matter how strong, may be epiphenomenal to aging.*

*Determining the underlying causes of these gene expression changes will require carefully designed future studies. We believe that at least some of these changes might originate from a systemic breakdown of homeostasis.*

*To acknowledge this in the manuscript, we added the following to Discussion: “... Second, it may be difficult to interpret the relationship between changes in gene expression and biological aging, as predicted by the ensemble method. Understanding mechanisms of aging remains an important challenge for biomedical research and our approach provides a tool to study aging in humans.”*

3. Then as far as I understand the authors test various methods to predict the classifiers and settle on one using cross validation. Then the authors emphasize the ability of their method to predict higher ages for progeria patients. However, I don't think progeria is universally accepted as accelerated aging, so such validation will not be convincing for everyone.

*The reviewer is correct that there has been a debate about whether progeria represents an accelerated aging phenotype. Accumulating evidence suggest that it is. One of the strong evidences is the mutation of envelope protein lamin A/C in progeria, and that age-related nuclear defects are caused by sporadic use of the same cryptic splice site in lamin A in healthy individuals (Scaffidi & Misteli, Science, 2006). While we are agnostic about this debate, our results are consistent with the hypothesis that progeria is a disease with an accelerated aging phenotype.*

*Nonetheless, we do acknowledge that this view may not be universally accepted, and thus we softened the text (3rd paragraph, p.4) to say, “HGPS provides a unique opportunity to study cellular processes in the context of what many believe is accelerated aging [15]”. We also removed the word “correctly” from the Abstract (i.e., “our method was the only one that correctly predicted accelerated aging in these patients.”), and similarly in Results.*

4. Related to the above, I also wonder about problems of overfitting, and whether other forms of validation would strengthen the work. For example, could the authors maybe split their data in half and then test how accurate the classifiers developed for half of the data are for the other half? Obviously, it would be ideal to have another data set the authors could apply their method to, but I'm not sure this exists.

*We agree that overfitting is always a worry. One machine learning technique that attempts to reduce overfitting is to use a training set to learn the model, and then a separate test set to validate the accuracy of the learned model. This is the approach we employed in our paper, under a leave-one-out cross-validation setting. Nonetheless, as suggested, we used a 50%-50% train-test split, with 10 different random shuffles of the data. The following results were obtained, which shows that the ensemble LDA continues to outperform the other methods: LDA (MAE=11.7 years, MED=5.0 years), Linear Regression (MAE=14.6 years, MED=13.1 years), Support Vector Regression (MAE=17.6 years, MED=15.1 years), and ElasticNet (MAE=18.0 years, MED=14.5 years).*

*These results are shown in Figure S2. We also inserted a brief note about these results in Methods: “In all cases, after grid search was used to select the best model parameters, model performance was evaluated separately using leave-one-out cross-validation to generate the results in Figure 1 and Table 1. As a further check against overfitting, we performed 10x random shuffles of 50%-50% training-test split (i.e., 2-fold cross-validation). The results, shown in Supplementary Figure S2, show that the ensemble LDA method continues to outperform the other methods on our dataset.”*

5. In the same paragraph the authors write: "This method predicts age from gene expression data in human fibroblasts more accurately than previous methods used to predict age from transcriptomic or epigenetic biomarkers [1-6]." Then write: "Our results are not far off the performance of Horvath's [1]". This seems like a contradiction, and overall I think the authors are overselling their findings in the discussion and should tone down their conclusions. For instance, the authors write that: "our method was the only one that correctly predicted accelerated aging in these patients." As far as I can tell the authors didn't test other methods, like epigenetic clocks, so I don't understand how can they make such claim.

*We thank the reviewer for highlighting this discrepancy, and we apologize for not clearly stating our meaning. Our intent was to say that our method predicts age more accurately than the regression algorithms used in other studies on our transcriptomic data. For instance, the DNA methylation (DNAm) clock uses ElasticNet regression (on a particular set of CpG sites), but ElasticNet did not perform as well as the Ensemble LDA on our gene FPKM data from fibroblasts. The reviewer is correct that we did not check the DNAm clock of the fibroblasts. To tone down our claims, we modified the text in three places: In 2nd sentence of the Discussion (p.5), we say: “Our method predicts age from gene expression data in cultured human fibroblasts more accurately than regression algorithms, such as ElasticNet, which have been previously used to predict age from transcriptomic, epigenetic, and other kinds of biomarkers [1–6].”*

*To further clarify this distinction between ElasticNet regression and the DNAm clock, we reworked the final sentence of that same paragraph, removing the statement “our method was the only one...” highlighted by the reviewer. This sentence now reads: “Furthermore, the ensemble method predicted HGPS patients as significantly older than age-matched controls, suggesting that the predictions made may reflect biological age rather than chronological age. In contrast, the regression algorithms did not make this prediction.” In the middle of that same Discussion paragraph we further toned down our comparison to other age prediction methods by adding the following: “This level of prediction performance using few samples is encouraging, yet the strength of the DNA methylation clock is the large numbers of studies and samples that have supported its use as an age predictor. While the learning curve (Figure 1B) suggests performance of our method will increase with additional samples, further work is required to test this.”*

6. I couldn't find information on how long and how many passages were cells cultured before the gene expression profiling.

*This information is in Supplementary Table S2.*

7. Please include page numbers in future manuscripts, to make it easier for reviewers to refer to specific sections in the manuscript.

*Fixed.*

**Reviewer #2**

1. What is the reason that different gene sets are used by different methods? By intuition, the same input (matrix of FPKM values for genes on the row and samples on the column) will make the comparison between methods on a fair basis.

*Thank you for raising this important question. Gene subsetting is only different for different folds of the leave-one-out cross-validation. It is not different across methods. Each method will be trained on the same 133 folds of leave-one-out cross-validation, and any individual fold will have the same sample composition (training data) and thus the same genes selected, no matter which method is being tested.*

*Because each fold of the cross-validation has a different sample composition (training data), there will be differences in what genes are selected for each fold. The idea is that every fold is treated as a unique dataset in order to avoid overfitting by selecting the genes based on all the training data. An alternative – to perform gene subsetting once using the entire dataset – could lead to information “leakage” because the held-out test data would be used to help select genes and thus train the model.*

*To ensure that readers understand this point we added the following comment to p.7, “Model fitting and evaluation”: “Note that gene subsetting is different across folds of the crossvalidation to prevent overfitting, but since the same folds will be used for training each model, gene subsetting is identical across models.”*

2. Why is the 20-year window better? Has the author examined other window sizes in addition to 10,20, and 30?

*It is difficult to derive a theoretical reason why the 20-year window is best. But intuitively, too small a window (bin size) means that there are too many classes and not enough data points within a class; too large a window means highly diverse ages would be grouped together and difficult to distinguish. When developing our method, we tried other window sizes. In Table 1, we reported 10, 20, and 30; below, as suggested, we also report 15, 25, and 35. We find the 20-year window performs best. We also added the following to the caption of Table 1: “Other window sizes (15, 25, 35) did not improve performance above that of the 20-year bin size.”*

Age bin size MAE MED R2

10 9.5 4 0.68

15 8.5 4 0.75

20 **7.7 4 0.81**

25 8.3 4 0.77

30 8.2 4 0.77

35 8.2 4 0.78

3. For the 22 test individuals in table 1, why aren't all other methods presented for this test?

*We have now added this material to Table 1.*

4. I suggest the authors also provide a scatter plot like Fig1A for the data in Fig1C. Maybe they

can arrange this figure in the supplementary.

*These plots are now available in Supplementary Figure S3.*

5. Why is LDA better? Although the authors mentioned "The efficacy of our ensemble method can be attributed to several features", that paragraph is not clearly explaining the "several features" and why those features make LDA better. Some further explanation is suggested but

not required or absolutely necessary.

*We apologize for the confusion. The paragraph the reviewer cites is attempting to explain why ensembles are effective in general, and to make those reasons concrete to the specifics of this age prediction problem and use of staggered age bins. To better explain this, that paragraph now reads:*

*“Why is the ensemble method so effective at predicting age from gene expression in fibroblasts? A necessary and sufficient condition for an ensemble to be more accurate than any of its individual classifiers is that those classifiers are accurate and diverse [18]. An accurate classifier has a lower error rate than uniform random guessing, and diverse classifiers make different errors when generalizing to new data. One taxonomy of ensemble learning [19] suggests that there are different ways to create classifier diversity, including manipulating the training examples (e.g., bootstrap aggregation) and the input features (e.g., sub-setting genes), or by injecting randomness (e.g., through random number generation for non-deterministic algorithms). Staggered age bins perform sample manipulation by grouping together different sets of subjects as belonging to the same age class for different classifiers of the ensemble. It is possible that this ensemble method with staggered discretizations could be generalized to other problems that involve prediction of smooth values within finite bounds.”*

*The next paragraph is the one that hypothesizes why LDA is an effective classifier to use in the ensemble. To further clarify our points this paragraph now reads:*

*“The ensemble with staggered age bins can be used with any kind of classification algorithm. LDA might be a particularly good choice for the ensemble because groups of genes involved in the same biological process are believed to co-vary in their expression. The LDA algorithm can exploit covariance structure to aid in classification, while its close relative naive Bayes assumes independence of the variables. Indeed, LDA had a mean error less than half that of naïve Bayes, which might reflect the interdependent nature of gene expression. While it is known that the transcriptional output of individual genes can change during aging [20], it is not yet clear if covariance patterns exist in gene expression programs over human lifespan [21]. Future work with our method may add to our understanding of how gene networks vary with age.”*

6. Not sure whether it is due to the big size of the dots in the Fig1A, there seem to be a large deviation for the ages between 50~70ish for the LDA method while other methods could more= accurately predict these ages. Could the authors explain this phenomenon? Is it possibly due to the different gene sets used for different methods? It's easy to decline a method if it predicts a 70-year old human being to be only 20-year old!! From these scatter plot, I would use LDA for most of the predictions but use the average of the other three methods if they are concordantly far away from the LDA prediction.

*The reviewer is correct that the ensemble LDA method does worse in the 70s than elsewhere in the age range. There is also increased error in the late 50s. However, inspection of Figure 1 shows that the other methods (ElasticNet, SVR, Linear regression) also perform the worst in the 70-85 age range. Thus, it does appear that, in general, these ages are the most difficult to predict. (As explained in our response to comment #1 above, this phenomenon is not due to different gene sets used for different methods.)*

*Why might this be? One explanation is that when you look at the sample density per half decade, it is clear that the 70s and late 50s have many fewer samples in them, as well as the highest mean error. While we previously reported the correlation between sample density and error rate, we now added Supplementary Figure S4 to make this correlation graphically clear. Thank you for helping us clear up this point.*

*Two additional points of note. First, as shown in Figure S4, while the ensemble LDA does have prediction errors in this age range that are > 50 years, the mean absolute error for the ensemble LDA in the 50-70 age range is still comparable to the other methods. That is, while the ensemble LDA is making some egregious errors, it is doing very well at other samples in the age range. Second, for each method, we reported both MAE (mean average error) and MED (median error) so that if a method reported a single 70-year old to be 20-years old, it would affect the MAE but not the MED as much. Finally, we tested the reviewer’s suggested idea of combining the different methods into a “meta-ensemble”. Specifically, we used the prediction of the ensemble LDA unless its prediction was more than X years away from the mean value of the predictions of the other 3 regression algorithms. For 34<X<41, we see a minor improvement in MAE (7.4 versus 7.7 using LDA alone, with the same MED error). For 30<X<=34 we see an even smaller improvement, and for values of X < 31 or X > 40, we see no improvement or a decline in performance compare to using LDA on its own. While marginal gains in error rate are possible by choosing which predictor to use in an ad hoc fashion, we believe that this procedure may be overfitting to the dataset, especially with regards to choosing the value of X, which likely does not generalize to other datasets.*

7. The authors did not explain why "The top expressed isoform was used as proxy for gene expression" while a common usage for gene expression to count all the reads on exons and compatible isoforms.

*Our method for quantifying gene expression is also commonly used as most genes express just one dominant isoform (for example, see PMC4053754) and is the default gene-level quantification used by the HOMER processing pipeline we employed. The benefit of this quantification method is that you avoid diluting the signal from exons that are not part of the main isoform being expressed. Further, our machine learning approach generalizes to any appropriate feature quantification method. We have added this explanation to the method section which now reads: "As most genes typically express just one dominant isoform, the top expressed isoform was used as proxy for gene expression.”*

8. Lastly, and most importantly, I think the authors should present the input FPKM matrices and downstream scripts for each prediction as additional files such that readers could learn the details of this manuscript.

*The software and FPKM tables are available to download as described in the manuscript section “Availability of data and material” on p.7.*

**Second round of review**

**Reviewer 1**

The authors have successfully addressed my concerns. The only thing they didn't answer was whether they provide the data on the genes used in the predictions, but this is a minor point.

**Reviewer 2**

The authors have significantly made improvements to the manuscript. They explained most of my questions. Although I am not perfectly satisfied, the explanations do seem acceptable with only one exception. That is question 7 regarding the use of the primary isoform to calculate the gene expression. They have provided only one article PMC4053754 to support this way of calculating gene expression. However that article was back in 2013, outdated. There's no explanation how dominant is so called dominant. Most of the citations to that article were discussing the opposite thing. Another thing is during aging process, the dominant transcript may well change from one isoform to another isoform. I think it is worthwhile for the authors to redo their analysis using all transcripts for one gene's expression. This may provide answers to the weird outlier in the predictions, such as those old age outliers.

**Authors Response**

**Point-by-point responses to the reviewers’ comments:**

Reviewer #1

1. The authors have successfully addressed my concerns. The only thing they didn't answer was whether they provide the data on the genes used in the predictions, but this is a minor point.

*We apologize for missing this. We anticipate that future studies will use our algorithm with larger datasets to further home-in on the genes most relevant for making accurate age predictions. Therefore, we added the following to the README file in the software repository on github to describe how users of our software can extract the genes used in the predictions:*

*After training a model, e.g. linregr, one might wish to examine the particular genes that meet subsetting criteria and that were used to predict age. This list of genes is in the class variable linregr.genecolumns. Similarly, one might wish to know how important a given gene is to creating the age prediction; this can be found in the class variable linregr.coef, which contains the linear coefficients that transform the FPKM vector (arranged in the order specified in genecolumns) into an age prediction for a given subject. For the ensemble, the genes used are in ensemble.genecolumns. The importance of each gene to an age prediction is more difficult to understand in an ensemble, since there are N classifiers in the ensemble, and each has coefficients for M = ceil( subject_age_range / N ) classes. The variable, ensemble.classifiers_[X].coef_[Y], where X ∈ [0,N) and Y ∈ [0,M), contains the linear coefficients that transform the FPKM vector into a confidence score that classifier X would assign this subject to class Y. A subject is assigned to the class with the highest such confidence score. For further details see the documentation and source code for LinearDiscriminantAnalysis in scikit-learn.*

*The software and data used in creating this paper are publicly available to download as described in the manuscript section “Availability of data and material” on p7. Included in the github repository is an implementation of our algorithm and the resulting trained regressions & ensemble stored in .npy files. By either loading these files or by re-running the analysis from scratch, one can see which genes meet subsetting criteria and were used to predict age. One can also look at the coefficients of each trained regression/classifier to assess how important each gene is for predicting age.*

Reviewer #2

1. The authors have significantly made improvements to the manuscript. They explained most of my questions. Although I am not perfectly satisfied, the explanations do seem acceptable with only one exception. That is question 7 regarding the use of the primary isoform to calculate the gene expression. They have provided only one article PMC4053754 to support this way of calculating gene expression. However that article was back in 2013, outdated. There's no explanation how dominant is so called dominant. Most of the citations to that article were discussing the opposite thing. Another thing is during aging process, the dominant transcript may well change from one isoform to another isoform. I think it is worthwhile for the authors to redo their analysis using all transcripts for one gene's expression. This may provide answers to the weird outlier in the predictions, such as those old age outliers.

We agree that isoform changes might be occurring as we age, but unfortunately our data and experimental design were not optimized for an isoform-level quantification. Specifically, below, we describe: (1) limitations of our data for performing isoform-level quantification; and (2) an analysis nonetheless of age prediction using all transcripts.

Limitations of our data for performing isoform-level quantification. There are many ways to quantify gene expression from sequencing data depending on the purpose of the analysis and the data quality. We optimized our choices to obtain gene-level counts in a large cohort of patients. To focus on isoform-level changes would require deeper sequencing, paired-end library preparation, and long-read sequencing (please see PMC4728800 for a general overview).

Indeed, when quantify isoform abundance using a well-established quantification pipeline, cufflinks (PMC3146043), we find that most isoforms cannot be reliably detected using our data. Most isoforms we quantified have a confidence range that is larger than the predicted abundance. To show this, we took the log2 normalized 95% confidence interval for all measured isoforms in all of our human samples, and divided by the relevant log2 normalized predicted FPKM value. This results in a normalized confidence range; the distribution of these confidence range values are shown in the boxplot in Fig A. While a few isoforms have confidence ranges that are much smaller than the actual predicted value (a y-value close to 0 in Fig A, inset), most isoforms have a normalized confidence range > 1, indicating that the confidence interval is large enough that the true expression level could be zero or much larger than the predicted value. Thus, the majority of isoforms cannot be reliably quantified using our data.

Fig A. A boxplot of the distribution of the normalized log2 FPKM isoform confidence ranges.

Cufflinks was used to predict isoform abundance (FPKM), and low/high 95% confidence intervals. Abundance and corresponding 95% confidence intervals were transformed to the log2 scale and normalized as: (high log2FPKM – low log2FPKM) / (log2FPKM). A confidence value of 1 indicates an estimated confidence range as large as the predicted FPKM abundance. The notch in the boxplot indicates the median of the distribution; the bar edges indicate 25th and 75th percentile; the whiskers show outliers. The vast majority of the distribution is well above 1, indicating that the majority of isoforms cannot be reliably quantified using our data. Inset shows zoom-in around the highlighted range.

Analysis of age prediction using all transcripts. Although the analysis above indicates that our isoform abundance estimates are low quality, we wish to address the reviewer’s concerns as best as we can with the data we have. Specifically, we re-trained the LDA ensemble and linear regression using transcript levels for all 182,657 isoforms quantified with the cufflinks cuffquant pipeline. Applying our previous gene subsetting criteria gave 38,811 isoforms of 12,090 unique genes as variables for prediction. Linear regression using 38,811 isoforms results in 13.3 years mean absolute prediction error, R2=0.71 using leave-one-out crossvalidation; in comparison the top isoform linear regression we reported in the paper used 4,861 genes and produced 12.1 years MAE, R2=0.73. This all isoform subset was too large for the ensemble, as calculating a 38,811 x 38,811 covariance matrix is computationally costly and actually exceeds a memory limit imposed by a software library we use (numpy.eigh). Therefore, we modified our gene subsetting criteria, and selected only isoforms for which at least 100 of the subjects had abundance > 5 FPKM and at least a 5-fold difference in FPKM existed between any two subjects. This resulted in 9,494 isoforms from 5,087 unique genes. The resulting ensemble produced a mean absolute prediction error of 8.6 years, R2=0.73 during leave-one-out cross-validation; in comparison the result in the paper using the top isoform was 7.7 years, R2=0.81. In summary, prediction with all isoforms is slightly worse than with top isoform, likely due to the increase in the number of variables without a concomitant increase in training samples.

Of note is that the general shape of the scatter plots from the manuscript’s Fig 1 remains qualitatively the same when using all isoforms instead of just the top isoform (Fig B). Just as when we used the top isoforms, using all isoforms results in about two dozen middle-aged “outlier” individuals with prediction error in the neighborhood of 20-60 years. 60% of the 20 worst-predicted subjects using the top isoform data are also in the 20 worst-predicted list using the all isoform data. The Pearson correlation between a subject’s prediction error with top isoform data vs. with all isoform data is 0.645. Finally, just as when we used top isoforms, using all isoforms results in progeria patients being predicted as older than age-matched controls. This time the progeria patients were predicted as 20.9 years older on average, whereas with top isoform they were predicted as 9.8 years older. In summary, using all isoforms to predict age does not change the nature of “outliers” in normal subjects, and similarly predicts progeria patients to be significantly older.

We thank the reviewer for suggesting rightly that isoform data may be of interest in addressing how gene expression changes with age; however, we do not feel that our current data can address this question in a useful and rigorous way. To include this briefly in the manuscript, we added to the section “RNA sequencing” in the methods: “While isoform-level quantification of expression may help better stratify age, our data is not able to address this question, as it would require deeper sequencing, paired-end library preparation, and/or long-read sequencing.”

Fig B. Results for the LDA ensemble with all isoform data, using 9,494 isoforms from 5,087

unique genes.

**Third round of review**

Reviewer 1:

I think the authors misunderstood my comments or I did not make myself clear. See below.

The author description, "As most genes typically express just one dominant isoform, the top expressed isoform was used as proxy for gene expression".

My comment, "I think it is worthwhile for the authors to redo their analysis using all transcripts for one gene's expression."

Suppose a gene has three isoforms A, B, and C with 300 reads uniquely mapped to A, 400 reads uniquely to B, 500 reads uniquely to C, and 200 reads to either A, B or C. From the author description, it seems the author are using 700 reads to represent this gene's expression because C is dominant (possibly 700 reads) compared to A (possibly 500 reads) and B (possibly 600 reads).

However I do not think that is a good or common strategy. There are two reasons. First, when there are two or more isoforms the dominant isoform is probably not very representative. In the above example the dominant isoform is using 700 reads but neglecting a significant amount of 700 reads from A and B. Second, in the old age, the dominant isoform C may not express at all while there may be still 1400 reads mapped to A and B. That may cause huge error.

That is why I want to see the result when all 1400 reads are used to represent the gene expression. So my phrase "using all transcripts for one gene's expression" in this example is simply translated as "using 1400 reads from 3 transcripts for this gene's expression in both young and old stage". I did not mean to change the feature from gene to isoforms in your model.

**Authors Response**

**Point-by-point responses to the reviewers’ comments:**

1. I think the authors misunderstood my comments or I did not make myself clear. See below. The author description, "As most genes typically express just one dominant isoform, the top expressed isoform was used as proxy for gene expression". My comment,

"I think it is worthwhile for the authors to redo their analysis using all transcripts for one gene's expression." Suppose a gene has three isoforms A, B, and C with 300 reads uniquely mapped to A, 400 reads uniquely to B, 500 reads uniquely to C, and 200 reads to either A, B or C. From the author description, it seems the author are using 700 reads to represent this gene's expression because C is dominant (possibly 700 reads) compared to A (possibly 500 reads) and B (possibly 600 reads).

However I do not think that is a good or common strategy. There are two reasons. First, when there are two or more isoforms the dominant isoform is probably not very representative. In the above example the dominant isoform is using 700 reads but neglecting a significant amount of 700 reads from A and B. Second, in the old age, the dominant isoform C may not express at all while there may be still 1400 reads mapped to A and B. That may cause huge error. That is why I want to see the result when all 1400 reads are used to represent the gene expression. So my phrase "using all transcripts for one gene's expression" in this example is simply translated as "using 1400 reads from 3 transcripts for this gene's expression in both young and old stage". I did not mean to change the feature from gene to isoforms in your model.

*To address the reviewer’s concerns, we re-ran the linear regression and 20-year ensemble LDA using the union of all exon reads for a gene, instead of just the reads mapping to the exons of the top isoform. Below we include Figure A comparing three forms of transcript quantification for age prediction: our original top-isoform method, the all-isoforms method (as described in our previous rebuttal), and the union-of-exons method. The union-of-exons method uses the same subsetting as the original top-isoform method presented in our paper. The top-isoform expression produced the best quantitative results. The overall shape of the scatter plots remains largely the same, and the mean average error (MAE) increased by between 0.1 and 1.9 years with all-isoform and union-of-exons as compared to the top-isoform results.*

*As the reviewer suggested, the union-of-exons method is more appropriate for accurately quantifying gene expression levels regardless of isoform. However, our top-isoform method, at least on this dataset, is more accurate for predicting age. One possible explanation for this may be that the ratio of isoforms changes with age while gene expression remains somewhat constant. Such an occurrence would be a valid signature of ageing, which would be eliminated by a union-of-exons quantification. We note that in this paper we are not trying to make statements about gene expression (in which case the reviewer’s proposed method is inarguably better), but rather we are seeking biomarkers of aging. We further note that since our fastq files are publicly available on GEO, anyone can explore alternative methods of quantifying gene expression on our dataset and use our quantification results as a benchmark.*

*Fig A. Comparison results from two age prediction algorithms (each row; linear regression and the 20-year ensemble LDA) using three methods of gene expression quantification (each column; top expressing isoform, all isoforms, and the union of all exon reads for each gene). Each panel shows the results of leave-one-out cross-validation predictions for 133 healthy individuals. Dots are plotted for each individual showing predicted age (y-axis) vs. true age (x-axis), with a line of best fit overlaid, and a shadow showing the 95% confidence interval of that line determined through bootstrap resampling of the dots. Text on bottom of each panel shows performance metrics of mean absolute error (MAE), median absolute error (MED), and R squared goodnessof- fit for the line of best fit. The dotted line is the ideal line, where true age equals predicted age.*

*Compared to other quantification methods, the top isoform method produces the lowest MAE for both prediction algorithms.*
